# Supplementary material for: PRAME-AS lncRNA, regulated by MZF1, modulates PRAME expression and cell stemness
Source: PLoS One. 2025 Sep 17;20(9):e0331190. doi: 10.1371/journal.pone.0331190 (PMC12443320; doi:10.1371/journal.pone.0331190)
Supplement: S1 Fig — Breast Cancer and Glioblastoma do not have normal samples. The data was derived from LinkedOmicsKB web tool (https://kb.linkedomics.org/). (PDF) [file pone.0331190.s001.pdf]

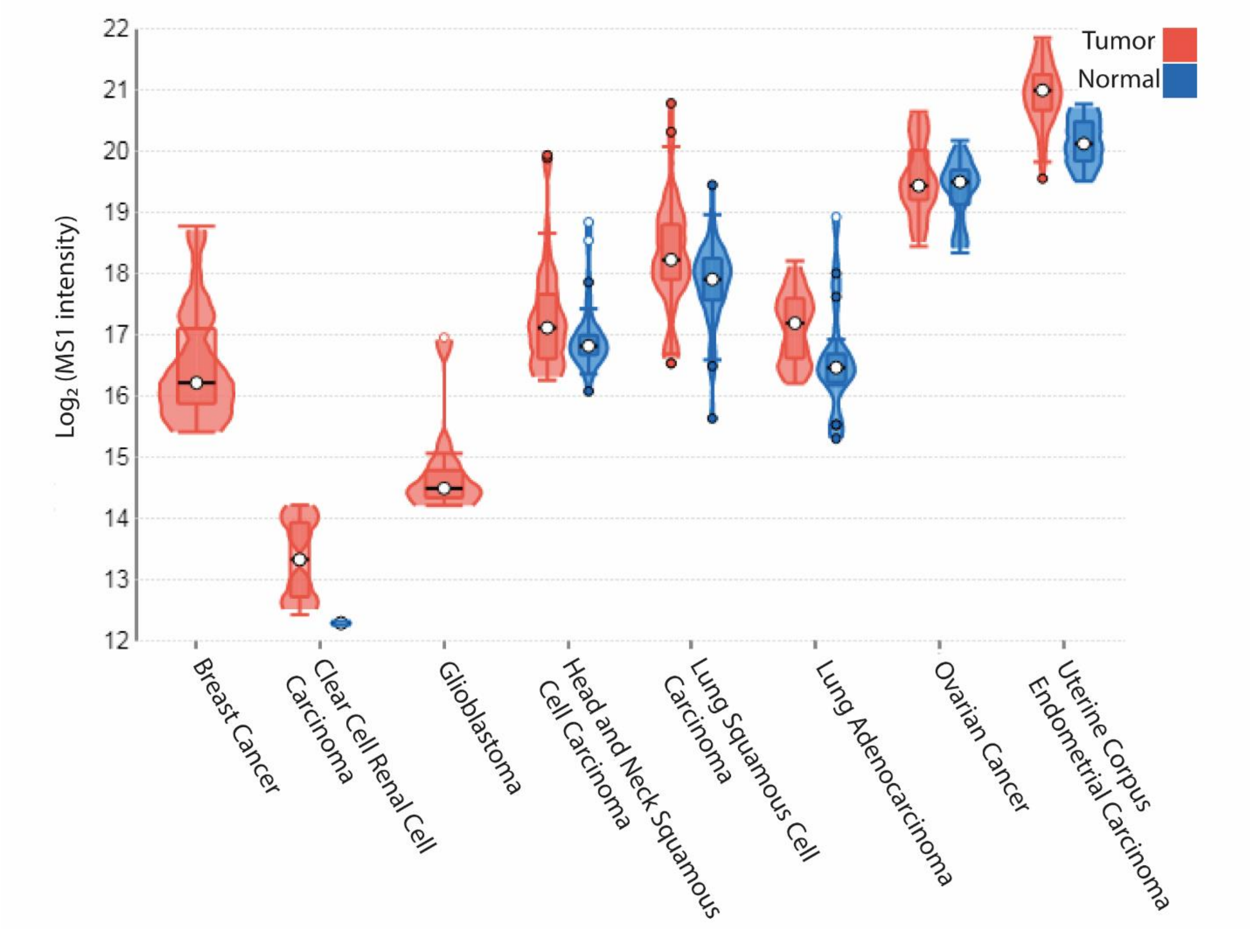

S1 Fig. Comparison of PRAME protein expression between tumor and normal tissues.

Breast Cancer and Glioblastoma do not have normal samples. The data was derived from LinkedOmicsKB web tool (**https://kb.linkedomics.org/**).
