# Supplementary figures and images for: PRAME-AS lncRNA, regulated by MZF1, modulates PRAME expression and cell stemness
Source: PLoS One. 2025 Sep 17;20(9):e0331190. doi: 10.1371/journal.pone.0331190 (PMC12443320; doi:10.1371/journal.pone.0331190)

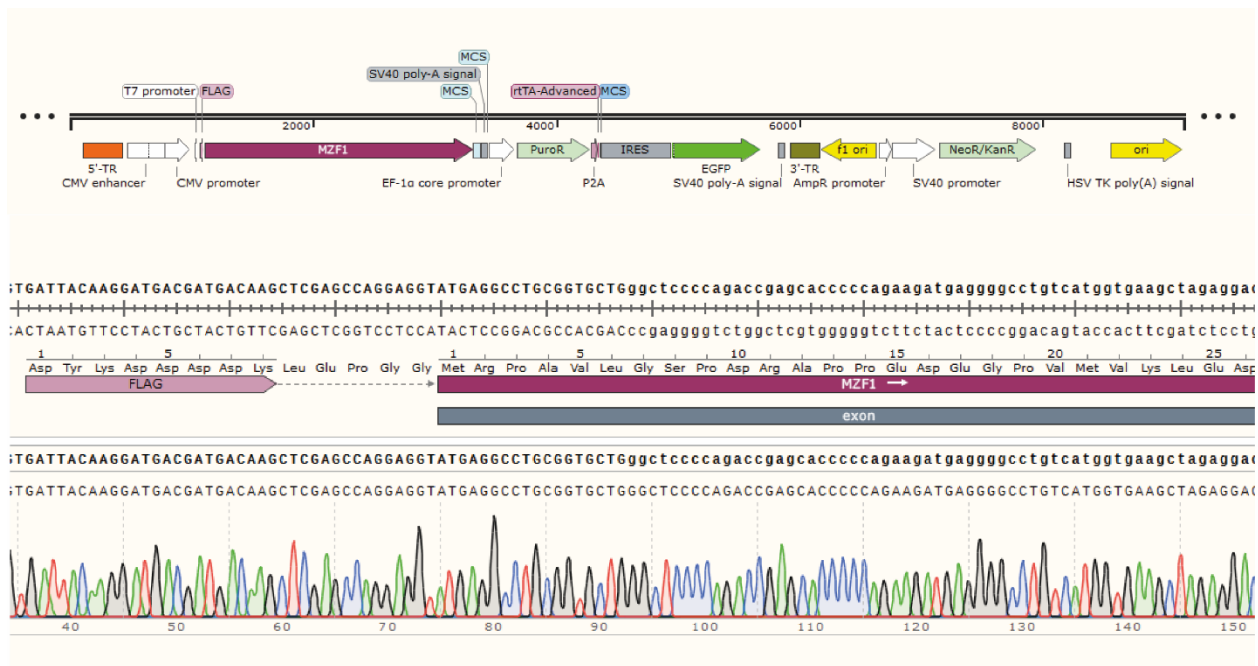

**S2 Fig. Map of the pHD\_4091 plasmid and the confirmation of the FLAG-MZF1 cloning by Sanger sequencing.**

Supplement: S2 Fig — (PDF) [file pone.0331190.s002.pdf]
