# Supplementary material for: PRAME-AS lncRNA, regulated by MZF1, modulates PRAME expression and cell stemness
Source: PLoS One. 2025 Sep 17;20(9):e0331190. doi: 10.1371/journal.pone.0331190 (PMC12443320; doi:10.1371/journal.pone.0331190)
Supplement: S6 Fig — The color of DMEM medium (A) and wound healing closure (B) in HEK293T cells exposed to different percentages of serum between two time points 0h and 24h after scratch (scale bar: 500 μm). (PDF) [file pone.0331190.s006.pdf]

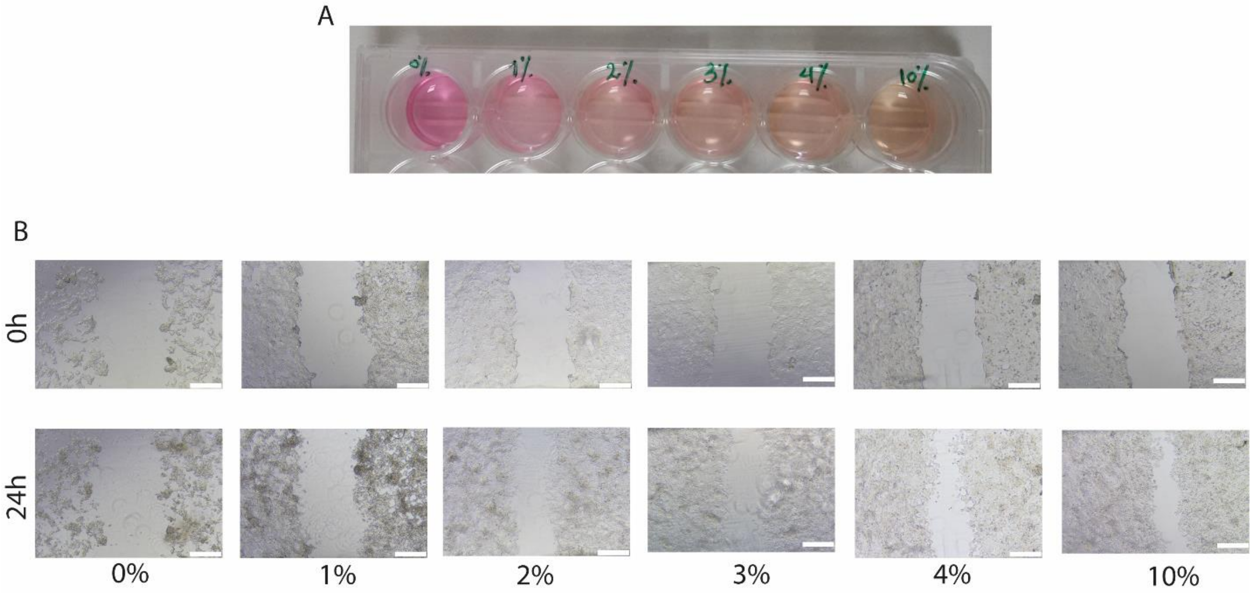

S6 Fig. Determination of the proper amount of serum in the DMEM medium for the scratch assay

The color of DMEM medium (A) and wound healing closure (B) in HEK293T cells exposed to different percentages of serum between two time points 0h and 24h after scratch (scale bar: 500 μm).
