# Supplementary material for: PRAME-AS lncRNA, regulated by MZF1, modulates PRAME expression and cell stemness
Source: PLoS One. 2025 Sep 17;20(9):e0331190. doi: 10.1371/journal.pone.0331190 (PMC12443320; doi:10.1371/journal.pone.0331190)
Supplement: S7 Fig — Dot plots compare the median transcript levels of PRAME (A) and PRAME-AS lncRNA (B) in tumor (red dots) and normal samples (green dots) from the TCGA and the GTEx databases, respectively. N: Normal, T: Tumor, n: Numbers. ACC; Adrenocortical carcinoma, BLCA; Bladder Urothelial Carcinoma, BRCA; Breast invasive carcinoma, CESC; Cervical squamous cell carcinoma and endocervical adenocarcinoma, CHOL; Cholangio carcinoma, COAD; Colon adenocarcinoma, DLBC; Lymphoid Neoplasm Diffuse Large B-cell Lymphoma, ESCA; Esophageal carcinoma, GBM; Glioblastoma multiforme, HNSC; Head and Neck squamous cell carcinoma, KICH; Kidney Chromophobe, KIRC; Kidney renal clear cell carcinoma, KIRP; Kidney renal papillary cell carcinoma, LGG; Brain Lower Grade Glioma, LIHC; Liver hepatocellular carcinoma, LUAD; Lung adenocarcinoma, LUSC; Lung squamous cell carcinoma, MESO; Mesothelioma, OV; Ovarian serous cystadenocarcinoma, PAAD; Pancreatic adenocarcinoma, PCPG; Pheochromocytoma and Paraganglioma, PRAD; Prostate adenocarcinoma, READ; Rectum adenocarcinoma, SARC; Sarcoma, SKCM; Skin Cutaneous Melanoma, STAD; Stomach adenocarcinoma, TGCT; Testicular Germ Cell Tumors, THCA; Thyroid carcinoma, THYM; Thymoma, UCEC; Uterine Corpus Endometrial Carcinoma, UCS; Uterine Carcinosarcoma, UVM; Uveal Melanoma. (PDF) [file pone.0331190.s007.pdf]

A

B

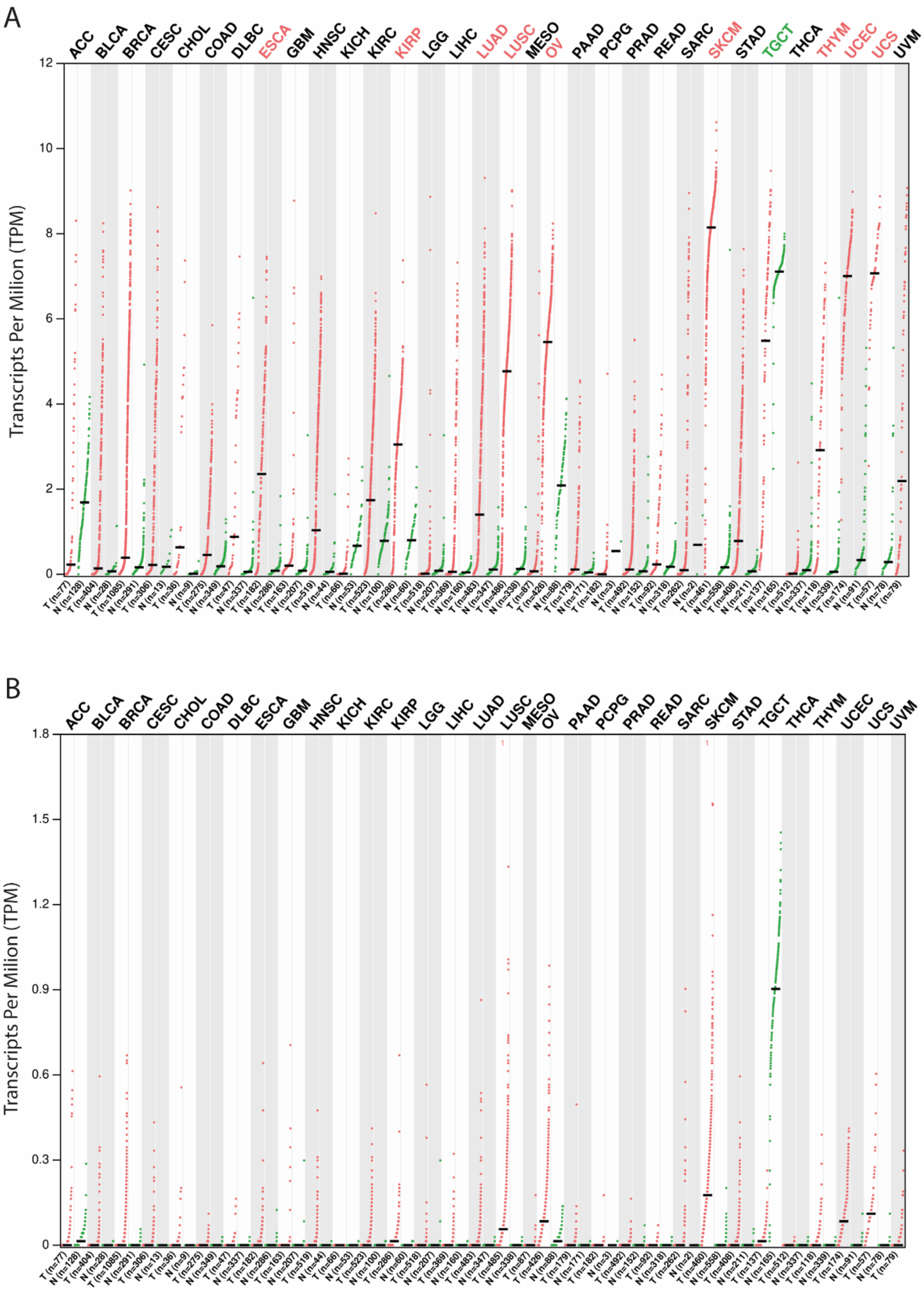
ACC
BLCA
BRCA
CESC
CHOL
COAD
DLBC
ESCA
GBM
HNSC
KICH
KIRC
KIRP
LGG
LIHC
LUAD
LUSC
MESO
OV
PAAD
PCPG
PRAD
READ
SARC
SKCM
STAD
TGCT
THCA
THYM
UCEC
UCS
UVM
Transcripts Per Milion (TPM)
0
2
4
6
8
10
12
0
0.3
0.6
0.9
1.2
1.5
1.8

S7 Fig. Comparative expression of PRAME and PRAME-AS lncRNA in normal and cancerous tissues

Dot plots compare the median transcript levels of PRAME (A) and PRAME-AS lncRNA (B) in tumor (red dots) and normal samples (green dots) from the TCGA and the GTEx databases, respectively.

N: Normal, T: Tumor, n: Numbers. ACC; Adrenocortical carcinoma, BLCA; Bladder Urothelial Carcinoma, BRCA; Breast invasive carcinoma, CESC; Cervical squamous cell carcinoma and endocervical adenocarcinoma, CHOL; Cholangio carcinoma, COAD; Colon adenocarcinoma, DLBC; Lymphoid Neoplasm Diffuse Large B-cell Lymphoma, ESCA; Esophageal carcinoma, GBM; Glioblastoma multiforme, HNSC; Head and Neck squamous cell carcinoma, KICH; Kidney Chromophobe, KIRC; Kidney renal clear cell carcinoma, KIRP; Kidney renal papillary cell carcinoma, LGG; Brain Lower Grade Glioma, LIHC; Liver hepatocellular carcinoma, LUAD; Lung adenocarcinoma, LUSC; Lung squamous cell carcinoma, MESO; Mesothelioma, OV; Ovarian serous cystadenocarcinoma, PAAD; Pancreatic adenocarcinoma, PCPG; Pheochromocytoma and Paraganglioma, PRAD; Prostate adenocarcinoma, READ; Rectum adenocarcinoma, SARC; Sarcoma, SKCM; Skin Cutaneous Melanoma, STAD; Stomach adenocarcinoma, TGCT; Testicular Germ Cell Tumors, THCA; Thyroid carcinoma, THYM; Thymoma, UCEC; Uterine Corpus Endometrial Carcinoma, UCS; Uterine Carcinosarcoma, UVM; Uveal Melanoma.
