# Supplementary material for: PRAME-AS lncRNA, regulated by MZF1, modulates PRAME expression and cell stemness
Source: PLoS One. 2025 Sep 17;20(9):e0331190. doi: 10.1371/journal.pone.0331190 (PMC12443320; doi:10.1371/journal.pone.0331190)
Supplement: S8 Fig — (A) Immunocytochemical detection of FLAG-MZF1 in the nucleus of MZF1-overexpressing cells (brown nuclei) (B) and the negative control cells (colorless nuclei) (Scale bar: 50 µm). (PDF) [file pone.0331190.s008.pdf]

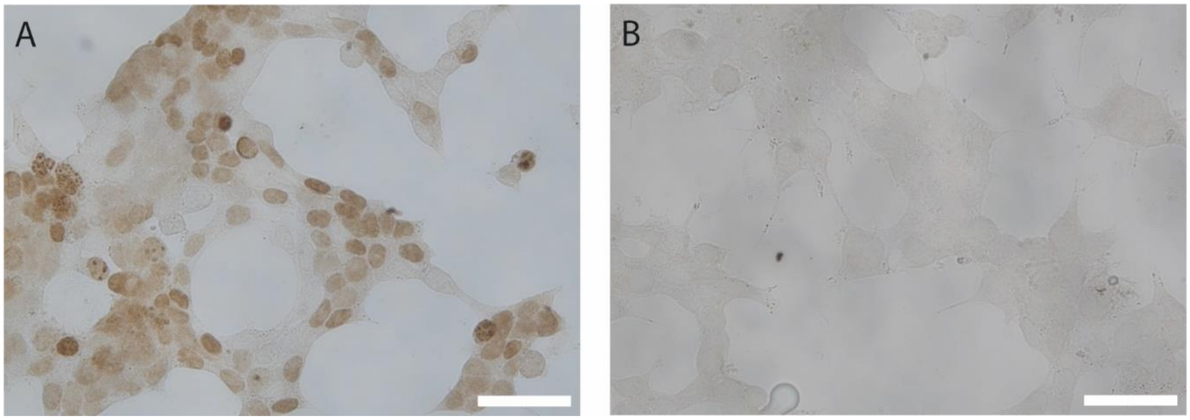

S8 Fig. Validation of the MZF1 protein expression in the MZF1-overexpressing cells.
(A) Immunocytochemical detection of FLAG-MZF1 in the nucleus of MZF1-overexpressing cells (brown nuclei) (B) and the negative control cells (colorless nuclei) (Scale bar: 50 µm).
