# Supplementary material for: PRAME-AS lncRNA, regulated by MZF1, modulates PRAME expression and cell stemness
Source: PLoS One. 2025 Sep 17;20(9):e0331190. doi: 10.1371/journal.pone.0331190 (PMC12443320; doi:10.1371/journal.pone.0331190)
Supplement: S9 Fig — The RNA copy numbers were quantified by RT-qPCR in three biological replicates. The RNA copy numbers for PRAME and PRAME-AS were normalized by the ACTB reference gene and reported as median with range. Statistical analysis was done using the Mann-Whitney U-test. (PDF) [file pone.0331190.s009.pdf]

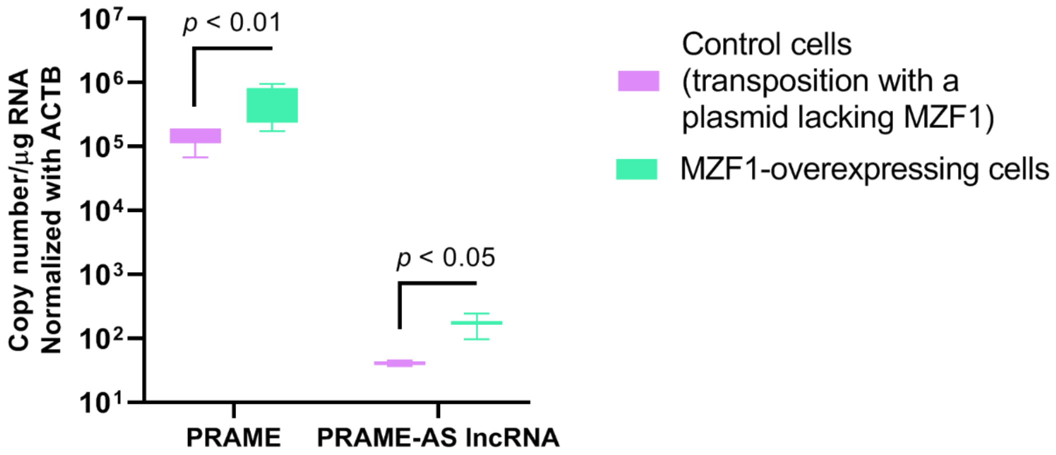

S9 Fig. Up-regulation of PRAME and PRAME-AS lncRNA transcript levels in the MZF1 overexpressing cells relative to the control cells. The RNA copy numbers were quantified by RT-qPCR in three biological replicates. The RNA copy numbers for PRAME and PRAME-AS were normalized by the ACTB reference gene and reported as median with range. Statistical analysis was done using the Mann-Whitney U-test.
