# Supplementary material for: PRAME-AS lncRNA, regulated by MZF1, modulates PRAME expression and cell stemness
Source: PLoS One. 2025 Sep 17;20(9):e0331190. doi: 10.1371/journal.pone.0331190 (PMC12443320; doi:10.1371/journal.pone.0331190)
Supplement: S1 Raw Images — A) The insertion location amplified by PCR. Lane 1: The PCR amplification of the third exon of the PRAME-AS lncRNA gene after insertion (using F2 and R2 primers) yields a 509 bp product. Lane 2: Restriction enzyme digestion of the 509 bp amplicon to confirm its sequence; 231 and 278 bp bands upon SacI digestion. Lane 3: A 50 bp DNA size marker (This image was used in Fig 2F). B) The RT-PCR products amplified from the insertion location. Lane 1: No template control (NTC), Lane 2: RT minus (minus-reverse transcriptase control), Lane 3: The RT-PCR amplification of the third exon of the PRAME-AS lncRNA transcripts after insertion (using F2 and R2 primers), expected to produce a 509 bp product. Lane 4: The PCR amplification of the third exon of the PRAME-AS lncRNA gene after insertion (using F2 and R2 primers) yields a 509 bp product as a positive control. Lane 5: The RT-PCR amplicon of ACTB (as a quality control for cDNA), Lane 6: A 50 bp DNA size marker (This image was used in Fig 2G). C) PCR amplicons for ACTB (lane 2; 257 bp), PRAME-AS (lane 3; 312 bp), and PRAME (lane 4; 212 bp). Lane 1; A 50 bp DNA size marker (This image was used in S5 Fig). In all cases, after electrophoresis, the agarose gels were stained by ethidium bromide. In order to visualize DNA bands, the stained gels were exposed to ultraviolet (UV) light. (PDF) [file pone.0331190.s010.pdf]

A

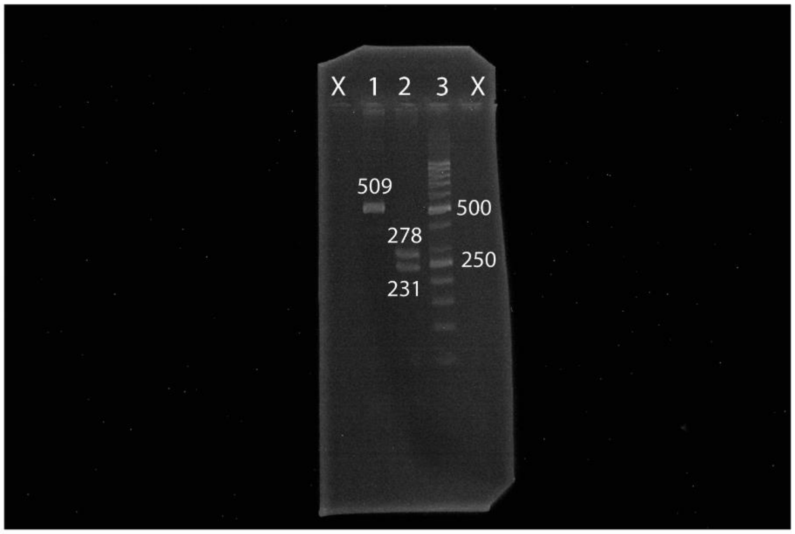
X 1 2 3 X
509
500
278
250
231

B

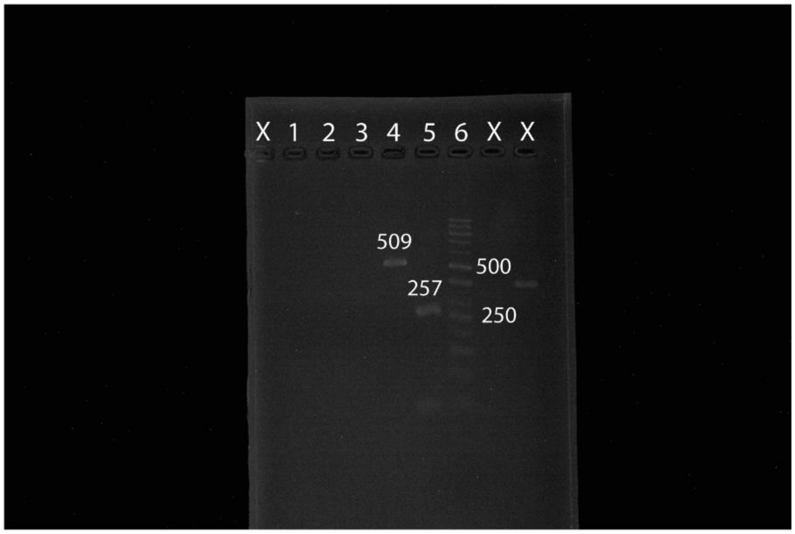
X 1 2 3 4 5 6 X X
509
500
257
250

C

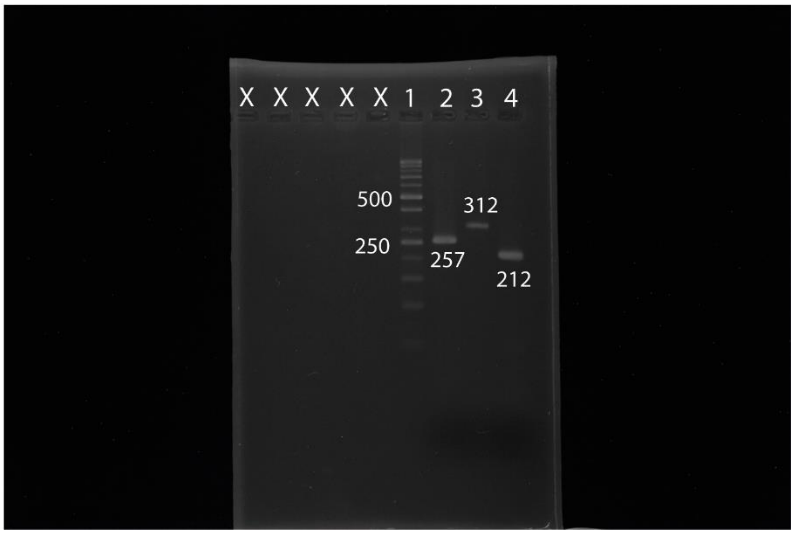
X X X X X 1 2 3 4
500
312
250
257
212

S1_raw_images. Agarose gel electrophoresis analysis of PCR products in this study.

A) The insertion location amplified by PCR. Lane 1: The PCR amplification of the third exon of the *PRAME-AS lncRNA* gene after insertion (using F2 and R2 primers) yields a 509 bp product. Lane 2: Restriction enzyme digestion of the 509 bp amplicon to confirm its sequence; 231 and 278 bp bands upon SacI digestion. Lane 3: A 50 bp DNA size marker (This image was used in Fig 2F).

B) The RT-PCR products amplified from the insertion location. Lane 1: No template control (NTC), Lane 2: RT minus (minus-reverse transcriptase control), Lane 3: The RT-PCR amplification of the third exon of the PRAME-AS lncRNA transcripts after insertion (using F2 and R2 primers), expected to produce a 509 bp product. Lane 4: The PCR amplification of the third exon of the *PRAME-AS lncRNA* gene after insertion (using F2 and R2 primers) yields a 509 bp product as a positive control. Lane 5: The RT-PCR amplicon of ACTB (as a quality control for cDNA), Lane 6: A 50 bp DNA size marker (This image was used in Fig 2G).

C) PCR amplicons for ACTB (lane 2; 257 bp), PRAME-AS (lane 3; 312 bp), and PRAME (lane 4; 212 bp). Lane 1; A 50 bp DNA size marker (This image was used in S5 Fig.).

In all cases, after electrophoresis, the agarose gels were stained by ethidium bromide. In order to visualize DNA bands, the stained gels were exposed to ultraviolet (UV) light.
