# Supplementary material for: PRAME-AS lncRNA, regulated by MZF1, modulates PRAME expression and cell stemness
Source: PLoS One. 2025 Sep 17;20(9):e0331190. doi: 10.1371/journal.pone.0331190 (PMC12443320; doi:10.1371/journal.pone.0331190)
Supplement: S1 File — (PDF) [file pone.0331190.s011.pdf]

S1 File: The 5’ to 3’ sequence of the cloned region of the PRAME locus regulatory region.

aagcaccgtactccgtgaagatgtgtgcagcctgttcccatgccctgaacaccatcccttcaagctcctctggctgctccctgctcaggtctcagaaaag
gagagctgggcaaccctcccagggagccgcccgcctgtgtaactttcagtaccagcctgtttgctttcttgagataattagctacaacggccactttaaa
acaatggtcttcttattttgtttttgttttcaccctaaactttataaagtcagaaatcgtaagtccttgagcatccacagccactcaaggacgaacctttg
ctgaatgcctcaagcagagagggggaggctgagtctctgtttcgaaggtgaaaacacaacggtttttatcagccctgttttatgcgacaatcccaggtc
gaatctggatcaatgcaatgacttcctttgcatggacctatgttttgttcgtgcattcatctgtgcagctgggaacccgcagctctgttcactggcatggg
gatgcctcggggaatggcagggcagagccacaaaaagccagcctcaccacgcggcctgttgctcttagccaccatgcccatcatagcctgtggcagg
agatgttggggtcccccccccgacccctttcaggaaaacagagtttgtggaggcggagtacttcccaggcctgactgataaaacaattcaaaagact
caaagagattcctcgcctgtcccgtcccttgaagggccttgaacacggggtccttccgcttggctcctagcccgtccctctccccttaccaggaaaggat
cctccccatctctgcagaagcctgaccatccccctagagggcctgggaggaagtgggttttgcatacagtccctgttgactctagtgccccctgctggcc
ccagacgcgagttccggcgaggcttcagggtacagctcccccgcagccagaagccgggcctgcagcgcctcagcaccgctccgggacaccccaccc
gcttcccaggcgtgacctgtcaacaggtctgtattggcgacaaaaggagcagccctgaatgtagggaaagcagggcggagtcctctgcaggctcggg
ggaggggaggggcgtgaatgcgtggatttctgtggagagtggaaacacggggagtcgaggggagcatgcgcgggcctcagaaagttctgggaaac
cgactcccgggagcagggaggaacgcgcgctccagagagtaagttaattgaatcccggggggtggtctcttcagcccgggggggttacagaagtaggt
aaatccagagattacccagccaaggccaggggcatcttttctagacttgcggggcggggtggggcgagaacttgagctc
