# Supplementary material for: PRAME-AS lncRNA, regulated by MZF1, modulates PRAME expression and cell stemness
Source: PLoS One. 2025 Sep 17;20(9):e0331190. doi: 10.1371/journal.pone.0331190 (PMC12443320; doi:10.1371/journal.pone.0331190)
Supplement: S1 Table — (PDF) [file pone.0331190.s012.pdf]

S1 Table: Details of HEK293T transfection for different applications of gene knockout, gene overexpression, CpG methylation, and PRAME locus regulatory region assay.

	Experimental groups	The amounts of plasmids[1]		The amounts of transfection reagent	The amount of puromycin for Selection	Duration of selection[2]
**knockout**	PRAME-AS knockout cells	5 µg/µl pHD_5009-1	5 µg/µl pHD_4012	2 µl of 1 mg/ml bPEI25[3]	0.7 µg/ml puromycin sulfate	3 days treatments 3 days recovery
**Overexpression**	MZF1 overexpressing cells	5 µg/µl pHD_4091	5 µg/µl pHD_3501	2 µl of 1 mg/ml bPEI25	0.6 µg/ml puromycin sulfate	3 days treatments 3 days recovery
	Control cell line (plasmid without FLAG-MZF1)	5 µg/µl pHD_4090	5 µg/µl of pHD_3501	2 µl of 1 mg/ml bPEI25		
**methylation**	dCas9-DNMT3A-treated cells	5 µg /µl pHD 71666_1	5 µg /µl pHD 71666_2	2 µl of 1 mg/ml bPEI25	No selection	-
	Control group (plasmids without sgRNAs)	10 µg/µl Addgene #71666		2 µl of 1 mg/ml bPEI25		
**PRAME locus regulatory region assay**	PRAME direction	10 µg of pHD4016-1		2 µl of 1 mg/ml bPEI25	No selection	-
	PRAME-AS LncRNA direction	10 µg of pHD4016-2		2 µl of 1 mg/ml bPEI25		
	Control group (promoterless plasmid)	10 µg of pHD4016-3		2 µl of 1 mg/ml bPEI25		

[1] Information of each plasmid is displayed in table 2
[2] The stage of puromycin treatment and recovery continued until we reached enriched EGFP expressed cell lines.
[3] 25 KDa branched polyethyleneimine
