# Supplementary material for: PRAME-AS lncRNA, regulated by MZF1, modulates PRAME expression and cell stemness
Source: PLoS One. 2025 Sep 17;20(9):e0331190. doi: 10.1371/journal.pone.0331190 (PMC12443320; doi:10.1371/journal.pone.0331190)
Supplement: Attachment — Submitted filename: Response to reviewers.pdf [file pone.0331190.s014.pdf]

RESPONSE TO REVIEWERS –(PONE-D-25-03086)

We would like to express our sincere gratitude to the reviewers for their time and thoughtful comments on our manuscript. Please find a point-by-point reply to the reviewers' comments below, and note that we have included the unedited comments of the reviewers in *italic*.

Reviewer 1

This study demonstrates that a lncRNA (PRAME-AS) can regulate its homologous coding gene PRAME. The authors identify MZF1 and hypermethylation of the upstream regulatory region as regulators of this lncRNA's transcription, and ultimately reveal that the PRAME promoter functions unidirectionally in regulating PRAME. While this discovery is novel, the main findings require further experimental validation to strengthen their impact.

Main concerns:

1. The title emphasizes the lncRNA and expression of PRAME, but the narrative lacks cohesion: The authors first identify the lncRNA, after knock out, the PRME expression is reduced, how does LncRNA regulate PRME? without explore further, the author turned to check cell proliferation, migration, viability, and anchorage-independent growth, then they turn to the explore its upstream regulatory factors (MZF1 and hypermethylation),and later shift to analyzing bidirectional promoter activity, Please ensure that all sections are connected naturally and smoothly.

Response: We thank the reviewer for this point. The main message of the manuscript is that PRAME-AS lncRNA can function as a regulator of PRAME transcript levels. We checked every instance regarding the relationship between these two genes throughout the manuscript and carefully corrected them to convey this message. Different sections of the manuscript that include these changes are listed below.

Title:
PRAME-AS lncRNA, Regulated by MZF1, Modulates PRAME Expression and Cell Stemness

Abstract:
Taken together, our data support the conclusion that PRAME-AS lncRNA acts as a regulator of PRAME transcript levels, and that MZF1—and the methylation status of a shared CpG island—influences the expression of both genes.

Introduction:
Overall, our data suggest that PRAME-AS lncRNA can be regarded as a regulator of PRAME transcript levels.

Results:
Subheading #2 and Figure 2 legend: Ablation of the PRAME-AS gene affects PRAME transcript levels

Discussion:
In this study, utilizing a knockout strategy, we demonstrate that the PRAME-AS lncRNA functions as a regulator of PRAME transcript levels (Fig 2).

This is the first study that investigates the PRAME-AS and the effects of its ablation on PRAME transcript levels (Fig 2).

We have edited the “Results” subheadings and added/changed explanations to provide a more cohesive narrative for this study.

2. The abstract uses non-definitive phrases ("lncRNA MIGHT regulate PRAME", "MZF1 MAY affect both genes"). If the results are reliable, replace tentative terms ("might/may") with stronger language (e.g.,"demonstrates" or "mediates") to enhance scientific rigor.

Response: We have corrected the abstract according to the reviewer’s point.

3. PRAME-AS lncRNA transcripts show no significant differential expression between normal and tumor tissues. This raises concerns about its functional role in tumor progression. what drives authors continues to explore this LncRNA in this situation?

Response: A lack of significant differential expression between normal and tumor tissues does not automatically undermine the functional relevance of an lncRNA. LncRNAs may exhibit crucial roles while not exhibiting differential expression. In addition, despite a subtle differential expression, changes in post-translational modifications, RNA stability, splicing patterns, cellular localization, or interactions with proteins could also influence their cellular functions.

4. The study attributes PRAME-AS-mediated phenotypes (proliferation, migration, etc.) to lncRNA activity, but it remains unclear whether these effects are PRAME-dependent or not. To confirm that the observed phenotypes are specifically regulated by PRAME-AS (and not solely by PRAME), include PRAME knockdown/knockout controls in functional assays.

Response: We thank the reviewer for this important point. It is very difficult—if not impossible—to directly relate the effects of a gene knockout to specific cell phenotypes (i.e., cell functions). The primary objective of our study was to knock out the antisense lncRNA gene and assess its impact on cell proliferation, viability, and stemness. We did not intend to attribute these effects to PRAME or PRAME-AS, nor have we stated anywhere in the manuscript that these phenotypes result from PRAME dysregulation.To clear this objective, we have changed and corrected the text throughout the manuscript accordingly:

Abstract:

The PRAME-AS knockout cells showed a decrease in migration, proliferation, stemness, and viability.

Results:

Subheading # 3 and Figure 3 legend: The PRAME-AS knockout cells showed a decrease in migration, proliferation, stemness, and viability

Minor points

1. Figure 1: Include PRAME protein expression to complement transcript-level findings. This could be added as supplementary data.

Response: A supplementary figure 1 (S1 Fig) containing the protein expression levels in different tumors has been added.

2. Figure 2G: Provide PRAME protein expression results following PRAME-AS knockout to validate transcriptional regulation at the protein level.

Response: We thank the reviewer for this point. We did not have access to the PRAME antibody. Thus, we kept our focus on the effects of PRAME-AS lncRNA on PRAME transcript levels.

3. Figure 4 Include Western blot data to confirm successful MZF1 overexpression.

Response: Since one of the defining features of MZF1 in a cell is its nuclear localization, and we have used these overexpressing cells in a separate study, we chose to perform immunocytochemical staining instead

of Western blotting to confirm MZF1 overexpression and its nuclear localization after transposon-mediated gene transfer. Our results (Fig 4 and S8 Fig.) showed that cells after transposition, compared to control cells, had strong nuclear signals.

4. Figure 6C Clearly the cell number of AS direction is much less than the other two.
Response: We thank the reviewer for this point. In Fig 6C, we replaced the panel of cells and used a better representative image.

Reviewer 2

In this paper, titled "PRAME-AS lncRNA is regulated by MZF1 and is involved in the expression of PRAME transcripts and cell stemness", the authors have undertaken a characterization of a previously unstudied lncRNA originating antisense from tumor antigen PRAME (PRAME-AS). Regulation of PRAME-AS has been analyzed via interrogation of known expression datasets, transcription factor overexpression, the efficacy of its promoter region, and the extent of CpG methylation surrounding it. The function of PRAME-AS was assessed via Cas9-mediated ablation with downstream impacts on PRAME expression, proliferation, and cell migration.

It is remarkable that for a gene with long known implications in tumorigenesis that the antisense lncRNA expressed at this locus has so far gone uncharacterized and so the authors should be commended for tackling this as a research topic. The method of PRAME-AS KO so as not to alter promoter activity of PRAME is elegant, however there are several concerns regarding the veracity of the conclusions until further clarity is reached.

Response: We sincerely appreciate the reviewer's insightful summary and positive evaluation.

Major comments:

Figure 2

1) What is the expression level of PRAME-AS in HEK293T cells, and how does it compare to PRAME expression approximately? Low expression levels are not necessarily an impediment to study as lncRNAs are commonly lowly expressed, but it should be made clear relative to the housekeeping gene.
Response: We have included the expression levels for PRAME-AS in wild-type cells. However, as we discussed and according to our previous and recent experiments, which have been incorporated in the "Results (Fig. 2F, Fig. 2G, and Fig. 2H)", the PRAME-AS transcript was not detectable in PRAME-AS knockout cells. We have added a new panel G to show that the PRAME-AS is not detectable by RT-PCR. We have also incorporated the expression level of PRAME-AS in wild-type cells to Panel H.

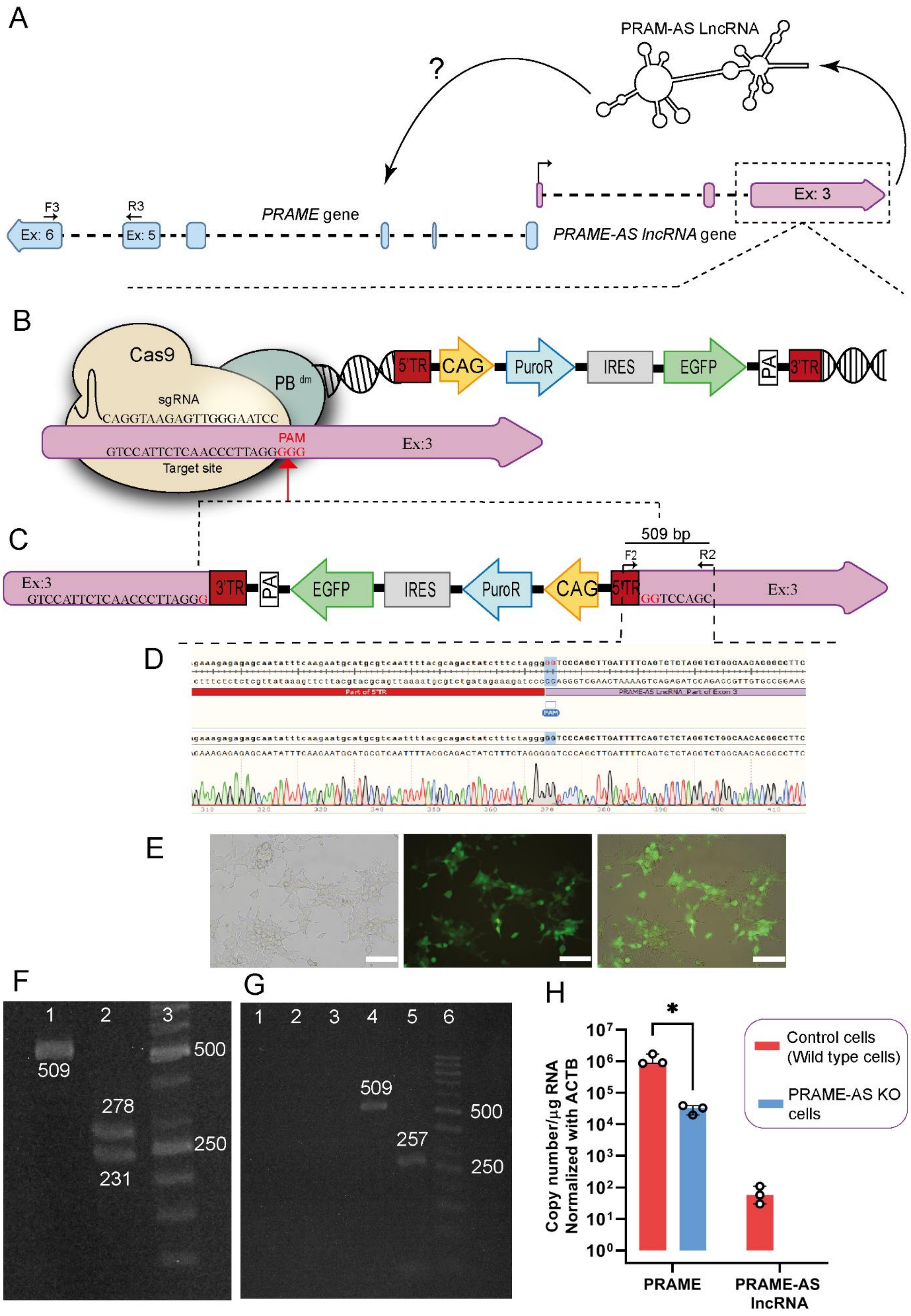
A
PRAM-AS LncRNA
?
F3
R3
Ex: 6
Ex: 5
PRAME gene
Ex: 3
PRAME-AS lncRNA gene
B
Cas9
PB dm
sgRNA
CAGGTAAGAGTTGGGAATCC
PAM
GTCCATTCTCAACCCTTAGGGGG
Target site
Ex:3
5'TR
CAG
PuroR
IRES
EGFP
PA
3'TR
C
509 bp
F2
R2
Ex:3
GTCCATTCTCAACCCTTAGGG
GGTCCAGC
D
E
F
1
2
3
509
278
231
500
250
G
1
2
3
4
5
6
509
257
500
250
H
Copy number/µg RNA Normalized with ACTB
Control cells (Wild type cells)
PRAME-AS KO cells
PRAME
PRAME-AS lncRNA
*

2) What is the extent of PRAME-AS decrease after integration of the donor fragment? Preferably shown with primers both upstream (Exon 2) and downstream (Exon 3) of the integrant.

Response: As explained in the previous answer, we were not able to detect PRAME-AS RNA after integration of the donor fragment Fig. 2G) when we used primers on either side of the integration point.

3) There is concern that inserting a construct inversely to PRAME-AS but on the same strand and upstream as PRAME may lead to interference with PRAME transcription if there is transcription readthrough of the construct.

Response: We thank the reviewer for this important point. In the original Figs 2B and 2C, we had mistakenly omitted the polyA signal. Now, the revised Fig. 2B and C show these signals, indicating that the transcription started from the CAG promoter will certainly stop at this point. In addition, between the end of the integrated transgene and the transcriptional start site of the PRAME, there is a 6,063 bp distance, which makes it very unlikely that the PRAME promoter would be affected by the transgene.

a) Does the sequence have (a) polyadenylation signal(s) to ensure transcription termination of the integrant? b) Can it be shown that there is no transcription readthrough downstream of the 3'TR region?

Response: The response to these questions has been provided in the above description.

4) Figure 2F: The presence of the Puro-IRES-GFP integrant is demonstrated, however it isn't clear or explicitly stated whether this is integrated into or one both alleles. Can this be shown or clarified?

Response: We thank the reviewer for this question. After integration of the CAG-Puro-IRES-EGFP-polA integrant, cells were subjected to several rounds of puromycin treatment. However, we did not grow cells clonally to be able to check them for mono-allelic or bi-allelic integration. We have incorporated a new "TransCRISTI method" section to Materials and Methods. The section is read as:

TransCRISTI method

To ablate the PRAME-AS lncRNA gene, we used our previously developed TransCRISTI method [28]. Briefly, HEK293T cells were transfected (using 25 kDa branched Polyethylenimine; bPEI25, Sigma-Aldrich, USA) with Cas9.PBdm (Cas9 fused to double mutant piggyBac transposase) and sgRNA #1 encoding plasmid (pHD_5009-1), and a donor plasmid (pHD_4012; 5' TR-CAG promoter-PuroR-IRES-EGFP-PA-3'TR) (Table S1). After 2 hours of incubation with polyplex, the transfection medium was replaced with the medium containing 10% FBS and no antibiotics. Forty-eight hours post-transfection, cells were selected for 3 days in the medium containing 0.7 µg/ml puromycin sulfate (Sigma-Aldrich, USA), followed by a 3-day recovery (medium without puromycin sulfate). Periods of puromycin treatment and recovery were continued until we had an enriched EGFP-expressing population of cells.

28. Rezazade Bazaz M, Ghahramani Seno MM, Dehghani H. Transposase-CRISPR mediated targeted integration (TransCRISTI) in the human genome. Sci Rep. 2022; 12(1):3390. PMID: 35232993.

Figure 3

1) It would be ideal to perform a rescue experiment of overexpressing PRAME in the PRAME-AS KO cells to determine whether those phenotypes are due to the ~34% loss of PRAME.

Response: We did not have access to a PRAME-expressing construct to perform this experiment. However, in several studies, as discussed in "Discussion", the PRAME overexpression has been linked to increased cell proliferation, cell stemness, and cell viability. In addition, as we explained in the answer to question 4 of Reviewer 1, we do not think that these cell phenotypes can all be related to the effects of a single gene (here, PRAME), and thus, overexpression (rescue) experiments and their effects on cell phenotypes would

not be ideal to study the effects of this knouckout further. Our primary objective was to demonstrate whether or not these cell functions are affected in response to the PRAME-AS knockout. We did not intend to label these effects as PRAME- or PRAME-AS-dependent. Related paragraph in "Discussion":

As previously indicated, there is strong evidence supporting the role of PRAME as a key contributor to cellular malignancy across various cancer types. For example, in triple-negative breast cancer (TNBC), PRAME overexpression induces the expression of 11 EMT (epithelial-to-mesenchymal transition)-related genes and leads to deregulation of Notch and Wnt signaling pathways, inhibition of apoptosis, and degradation of basement membranes in blood vessels [8]. Moreover, PRAME is elevated in hepatocellular carcinoma and prohibits apoptosis mediated by p53 and Bcl2 [9]. High level of PRAME has a strong positive correlation with the EMT-related transcription factors SNAI1 and TWIST, while normal liver regulators, including FOXA2 and AR, are negatively correlated with PRAME expression [26]. PRAME promotes the proliferation of osteosarcoma cells [10], while in Chronic myelogenous leukemia, in the presence of RA and PcG proteins, including EZH2, it binds to retinoic acid receptor (RAR) to block Caspase 3, TRAIL, and P21 expression, leading to proliferation and apoptosis arrest [14,15]. Another study in seminomas indicates that the overexpression of PRAME and its interaction with Lin28A support pluripotency networks and expression of reprogramming markers, such as DPPA3, Nanog, Oct3/4, ZSCAN10, and PRDM14 [20]. Two distinct research reports on non-small cell lung cancer (NSCLC) patients of Asia (South Korea, Singapore, Taiwan, and Thailand) showed a higher rate of PRAME transcripts in squamous cell carcinoma (SCC), and in smokers compared with non-smokers [16,17]. A recent study on cervical cancer indicates PRAME's involvement in proliferation, invasion, migration, reduction of apoptotic cells, and facilitating G0/G1 cell-cycle by the Wnt/β-Catenin pathway [21]. Another study supports the role of PRAME in proliferation, invasion, migratory potential, and EMT in LSCC, and the growth of tumors in response to PI3K/AKT/mTOR pathway activation [27]. These reports highlight the involvement of diverse genetic and epigenetic factors, regulatory networks, and pathways downstream of PRAME that influence cellular functions such as proliferation, viability, and stemness. In this study, we expand the players to add the PRAME-AS lncRNA to this regulatory network. However, since in this study, we manipulated only the PRAME-AS gene, we cannot expect to observe considerable changes in all kinds of cellular functions (Fig 3). For example, in the PRAME-AS knockout cells, MZF1, which is a positive regulator of PRAME, is still expressed and will continue to play its regulatory role.

Figure 4

1) Related to Figure 2, the fold change of PRAME and PRAME-AS can be seen but how do they compare to each other? It would be very interesting to see similar fold increases when overexpressing MZF1 if there were a large basal difference between PRAME and PRAME-AS.

Response: To show the basal difference between PRAME and PRAME-AS transcript levels, we included a supplementary S9 Figure, which has been prepared based on the copy number.

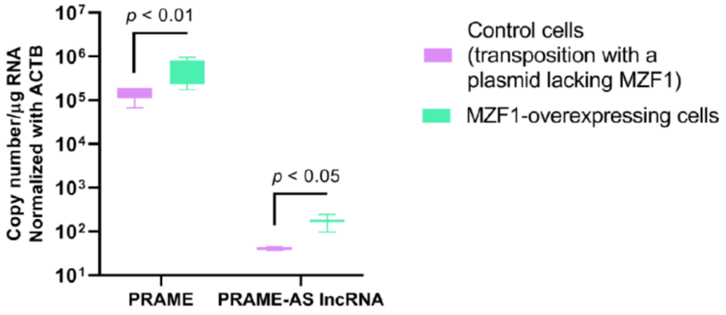

S9 Fig. Up-regulation of PRAME and PRAME-AS lncRNA transcript levels in the MZF1 overexpressing cells relative to the control cells. The RNA copy numbers were quantified by RT-qPCR in three biological replicates. The RNA copy numbers for PRAME and PRAME-AS were normalized by the ACTB reference gene and reported as median with range. Statistical analysis was done using the Mann-Whitney U-test.

Figure 5

1) If PRAME-AS expression levels are already low, and methylation levels high, then any dCas9 methylation will likely not alter PRAME-AS expression levels further. Can the level of methylation of each of the CpGs analyzed with bisulfite sequencing be quantified?

Response: To answer this question, we analyzed the methylation status of CpG dinucleotides in the HEK293T wild type (before transfection) and added this result to Fig. 5D.. As the sequencing results show, the CpGs were methylated; however, after utilizing the dCas9-DNMT3A, the amount of methylation was increased.

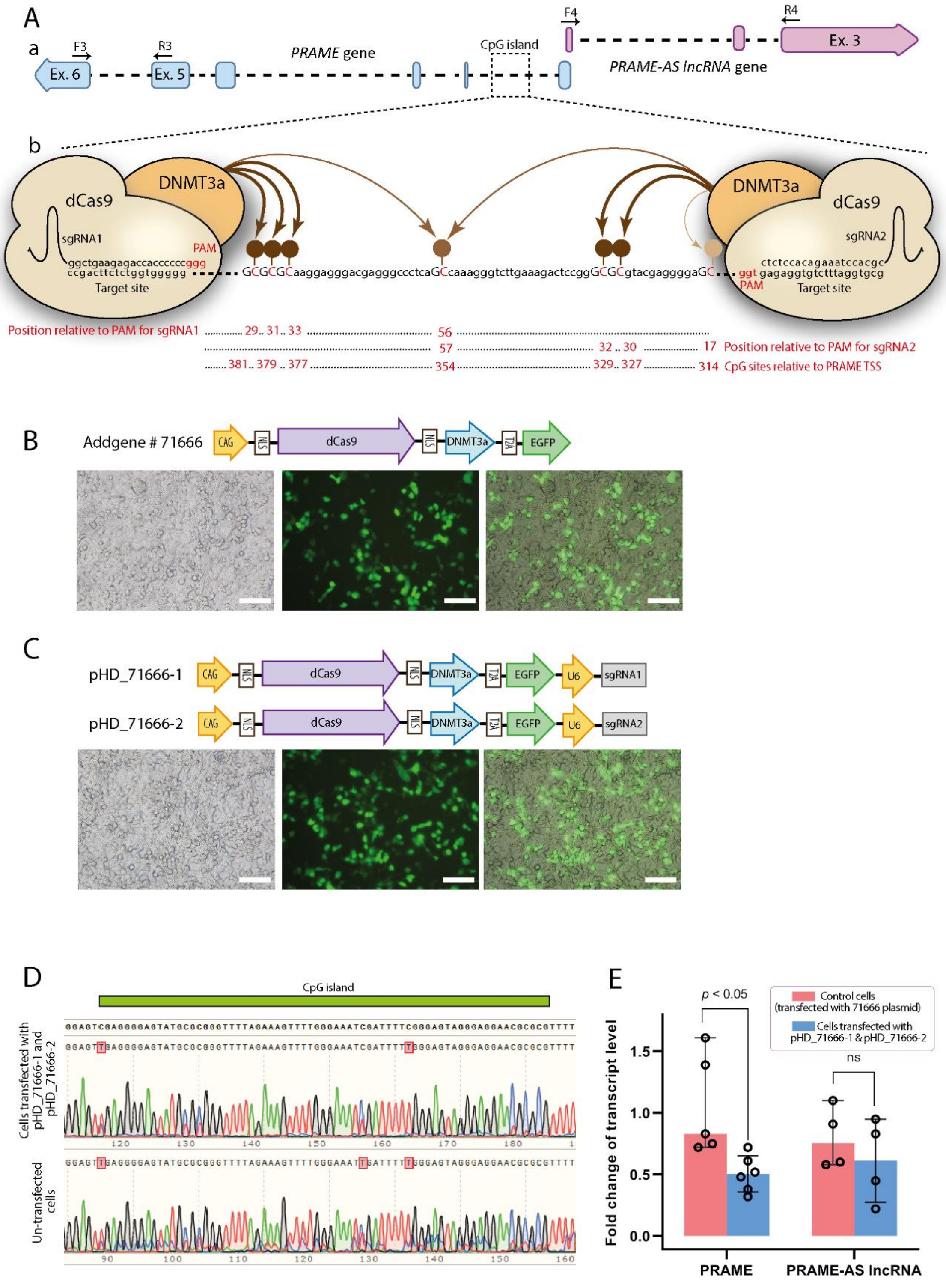

2) The images look as though transfection efficiency is not 100%, which likely dampens the impact on expression differences between negative control and dCas9-DNMT1 plasmids, which may be especially relevant if the levels of PRAME-AS are already low. Can the GFP+ cells be enriched via cell sorting to parse this out, or can the authors speak to this possibility?

Response: We appreciate this point. This is a valid explanation that untransfected cells may dampen

differences between two groups of cells. We did not have access to a cell sorter to be able to separate cells. However, by sequencing the CpG island, we checked the methylation status of the region and made certain that the CpG methylation had been affected after transfection.

3) The title of the figure states that hypermethylation diminishes PRAME-AS transcripts yet the quantification in Figure 5D is not significant, this should be corrected or clarified.

Response: We agree. We have changed the Figure 5 title and the corresponding "Results' subheading accordingly. Certainly, the lncRNA levels are affected, but not in a statistically significant fashion.

The new title reads as:

Figure 5. Hypermethylation of a regulatory region in the PRAME locus affects PRAME and PRAME-AS lncRNA transcripts

Previous Figure 5 title: Figure 5. Hypermethylation of a regulatory region in the PRAME locus diminishes both PRAME and PRAME-AS lncRNA transcripts

Figure 6

Here the authors are attempting to determine whether PRAME-AS is the result of a bidirectional promoter (from PRAME). Here, the luciferase results from Schenk et al (2007) are used as a marker for determining where the maximum promoter activity for PRAME is located. In that paper, PRAME is shown to have two transcription start sites (TSS) annotated, in which the downstream one has stronger activity, and may be the source of the bidirectionally of PRAME-AS, thus the overlap of the 5' exon is due to annotation of the upstream PRAME TSS with the antisense transcription of PRAME-AS resulting from the downstream PRAME TSS. Can the authors speak to this possibility?

Response: Yes, it is possible that the second (the downstream) TSS would have stronger effects on the PRAME-AS TSS. We have cloned the region in a way that includes both TSS sites. The revised Fig 6A shows these sites as parts of the cloned region. We have added this point to "Results":

The HEK293T cells were transiently transfected with the EGFP reporter constructs, containing a 1361-bp sequence (PRAME locus regulatory region) as promoter (containing two annotated TSS; [22]) in two opposite directions (Fig 6B).

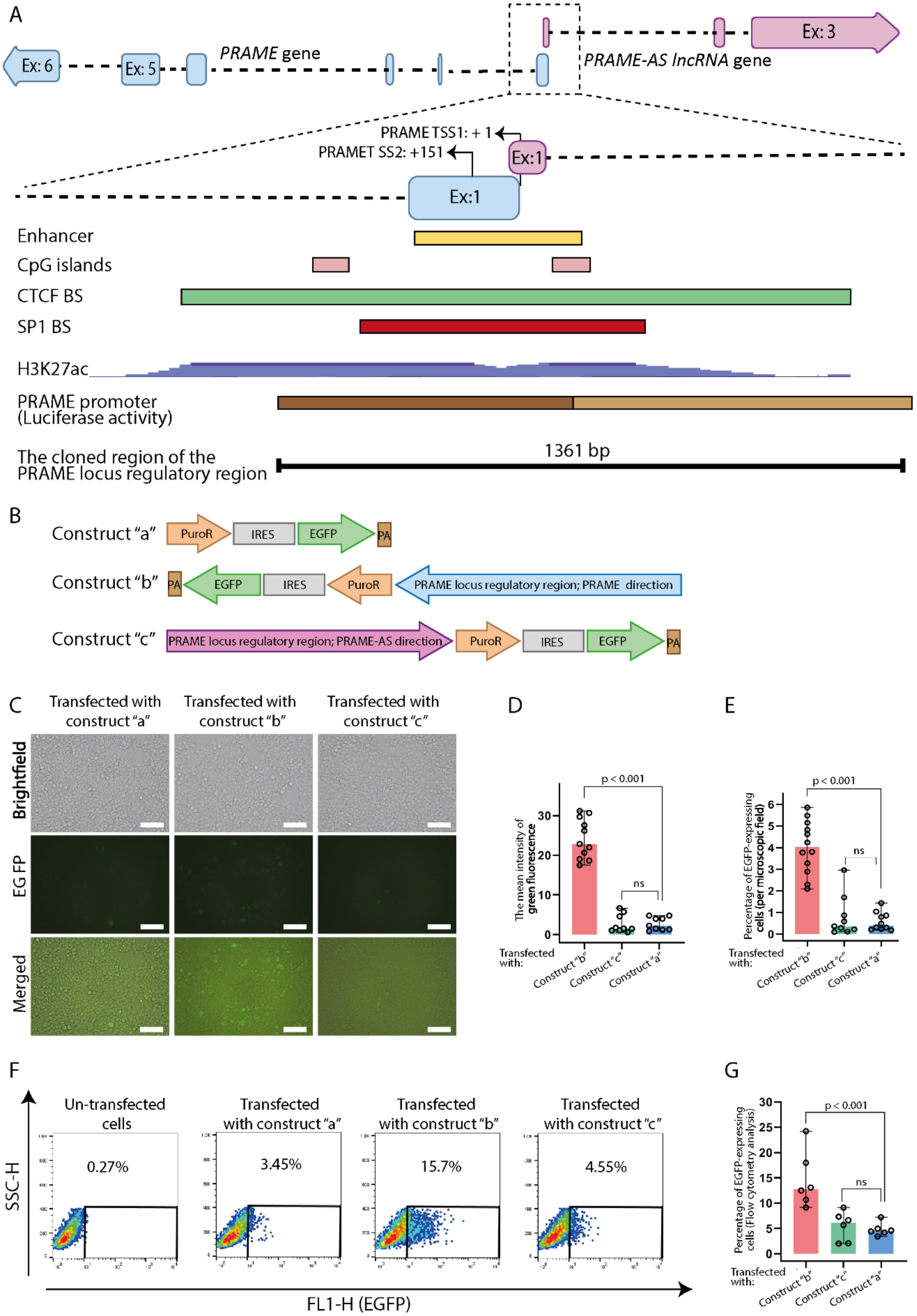
A
Ex: 6
Ex: 5
PRAME gene
Ex: 3
PRAME-AS lncRNA gene
PRAME TSS1: + 1
PRAMET SS2: +151
Ex:1
Ex:1
Enhancer
CpG islands
CTCF BS
SP1 BS
H3K27ac
PRAME promoter
(Luciferase activity)
The cloned region of the
PRAME locus regulatory region
1361 bp
B
Construct "a"
PuroR
IRES
EGFP
PA
Construct "b"
PA
EGFP
IRES
PuroR
PRAME locus regulatory region; PRAME direction
Construct "c"
PRAME locus regulatory region; PRAME-AS direction
PuroR
IRES
EGFP
PA
C
Transfected with construct "a"
Transfected with construct "b"
Transfected with construct "c"
Brightfield
EGFP
Merged
D
The mean intensity of green fluorescence
p < 0.001
ns
Transfected with:
Construct "b"
Construct "c"
Construct "a"
E
Percentage of EGFP-expressing cells (per microscopic field)
p < 0.001
ns
Transfected with:
Construct "b"
Construct "c"
Construct "a"
F
Un-transfected cells
Transfected with construct "a"
Transfected with construct "b"
Transfected with construct "c"
0.27%
3.45%
15.7%
4.55%
SSC-H
FL1-H (EGFP)
G
Percentage of EGFP-expressing cells (Flow cytometry analysis)
p < 0.001
ns
Transfected with:
Construct "b"
Construct "c"
Construct "a"

Bidirectional transcription from protein-coding genes often contain promoter proximal polyadenylation signals in the antisense direction (in this case PRAME-AS). Can the authors confirm whether the "PRAME-AS direction" construct contains any AATAAA/ATTAAA signals that may prematurely terminate transcription upstream of GFP?
Response: We checked the sequence of the cloned region and did not find any of these signals. Now, we have included the complete sequence of the cloned regulatory region in the supplementary file 1.

A much more convincing demonstration of bidirectional promoter activity would be to only include the CpG island region as shown in Figure 5 (perhaps overlapping PRAME promoter as well), if possible even including +/- in vitro construct methylation by SssI as shown in Shenk et al. Alternatively, can it be explained in more detail as to how this region is not "bidirectional" based on the tests that have already been completed?
Response: We agree that our previous explanation was not clear. We have corrected the Results (subheading title, and the relevant text, and Fig. 6). Our explanation now clearly indicates what the experiment was intended to show. The brief response is that we checked whether the predicted regulatory region, in two opposite directions, is able to activate the transcription of a downstream EGFP reporter. And, what we found was that this regulatory region was able to activate the EGFP reporter transcription only in the PRAME direction.

Our revised Results subheading and the relevant text clearly explain this experiment:

The PRAME locus regulatory region regulates transcription of a reporter gene only in the PRAME direction
PRAME and PRAME-AS genes are located in a head-to-head configuration with overlapping 5' ends (Fig 6A). This feature, along with two CpG islands, numerous SP1 binding sites in addition to other signs of active transcription, such as H3K27ac (open chromatin signals) and enhancer sequence, might indicate the presence of a bidirectional promoter, working for both genes (Fig 6A). The HEK293T cells were transiently transfected with the EGFP reporter constructs, containing a 1361-bp sequence (PRAME locus regulatory region) as promoter (containing two annotated TSS; [22]) in two opposite directions (Fig 6B). We observed reporter activity only in those cells that had the construct in the PRAME direction (Fig 6C-E). Flow cytometry analysis also displayed that the percentage of EGFP-expressing cells in the PRAME direction (14.6%) was more than that in the promoterless vector (4.81 %) (Fig 6F, G). However, the PRAME promoter in the opposite direction could not induce significant expression of the EGFP protein (5.47 %) (Fig 6F, G). These results indicate the unidirectionality bias of the PRAME promoter, functioning in the PRAME direction.

Minor Comments

1) Figure 1: Font of y-axis is very small, should be enlarged to scale with the rest of the graph
Response: We have corrected the y-axis in Fig. 1.

2) Figure 1: It is very interesting that PRAME-AS appears to be completely silenced in testicular germ cell tumors. What is the p-value of the comparison between testicular germ-cell tumors and normal testicular cells
Response: For both PRAME and PRAME-AS, the *p-value* was set as the default p < 0.01 in the GEPIA2 web server. We have added this explanation to the Fig. 1 legend.

3) It is fine to reference a prior paper for details on the methods regarding TransCRISTI, however the methods section of this publication should still have a section dedicated to it, even if it is only to refer to the prior paper.
Response: As we explained in response to the question on Fig. 2, we added a section on the TransCRISTI method to "Materials and Methods".

4) Figure 5: Can it be labeled more explicitly which sgRNA corresponds to which CpG island?
Response: Now, we have changed the labeling of CpGs and corresponding sgRNAs in Fig. 5 and have added descriptions in the legend. We also added a description in "Materials and Methods" to clearly show how the target sequences for these sgRNAs have been selected.

Materials and Methods:

In order to induce methylation of a CpG island in the regulatory region of the PRAME-AS gene, we mapped the exact position of predicted CpG dinucleotides according to Schenk et al. [22]. In the next step, two sgRNAs were designed to target dCas9-DNMT3A molecules to this island. According to Vojta et al. [30], a 25-35 bp region downstream of the protospacer adjacent motif (PAM) can be an optimum range for an effective methylation induced by dCas9-DNMT3A. And, the CpG dinucleotides outside the optimum range of 25-35 bp from the PAM sequence are less likely to become methylated. …

Fig. 5 legend:

Ab) Schematic illustration of dCas9-DNMT3A effectors targeting a CpG island using two specific sgRNAs that bind 25– 35 bp downstream (the effective range for a fully methylated state) of the PAM sequences [30]. The position of CpG dinucleotides is shown relative to the nearest sgRNA and to the PRAME transcriptional start site. The thickness and higher color intensity of the brown arrows correspond to the higher DNMT3A activity.

5) Figure 4: What happens to PRAME and PRAME-AS expression if MZF1 is upregulated in PRAME-AS KO cells? Is there a link between MZF1 recruitment and PRAME-AS influence on PRAME?
Response: We did not perform this experiment. MZF1 binds to the upstream regulatory sequences of the *PRAME-AS* gene (Fig. 4A). After the insertion of the targeting fragment in the Exon 3 of the *PRAME-AS* gene, the lncRNA transcript becomes undetectable. Thus, upregulation of the *PRAME-AS* gene in PRAME-AS KO cells seems unlikely. Although not analyzed in this study, we expect that following MZF1 overexpression, the PRAME transcript levels will be increased in the PRAME-AS KO cells.

6) Figure 5: Could the authors show that reducing DNA methylation at the PRAME-AS 'promoter' leads to increased PRAME-AS expression i.e through dCas9-Tet1?
Response: Unfortunately, we do not have access to a dCas9-Tet1 construct to be able to perform this experiment.

Reviewer 3:

Overview

In this study, the authors investigate the effects of long non-coding RNA (LncRNA) PRAME-AS gene on the expression of PRAME, cell proliferation, migration, cell viability and anchorage-independent growth with results showing a decrease in all of these functions. Upregulation of PRAME-AS via MZF1 overexpression and hypermethylation lead to an increase in the above-mentioned characteristics.
Overall, this study was written clearly and the work was well performed.

Response: We sincerely appreciate the reviewer's positive evaluation.

Comments

Major comments:

1. A full description of the TCGA and GTEx datasets for bioinformatics analyses should be included in M&M.

Response: We used the GEPIA2 online tool that exploits the data of the TCGA and GTEx datasets. In the revised version, we have included a description in Materials and Methods and explained the analyses on PRAME and PRAME-AS lncRNA transcripts and PRAME protein levels.

2. Some of the results sections describe details of the technique used more than an elaborate description of the actual results and their significance.

Response: We appreciate the reviewer's feedback on this point. We reviewed the "Results" and omitted all detailed technical descriptions that were already described in "Materials and methods".

3. More focus on the significance of the findings is needed in the discussion.

Response:

We agree, and we thank the reviewer for this point. In the revised version, we have added the following sentences to "Discussion" to emphasize the importance of our results.

Further investigation is required to elucidate the precise mechanisms by which PRAME-AS regulates PRAME expression. These regulatory mechanisms may involve direct interactions between PRAME and PRAME-AS, such as the formation of an RNA–RNA duplex.

These reports highlight the involvement of diverse genetic and epigenetic factors, regulatory networks, and pathways downstream of PRAME that influence cellular functions such as proliferation, viability, and stemness. In this study, we expand the players to add the PRAME-AS lncRNA to this regulatory network.

However, since in this study, we manipulated only the PRAME-AS gene, we cannot expect to observe considerable changes in all kinds of cellular functions (Fig 3). For example, in the PRAME-AS knockout cells, MZF1, which is a positive regulator of PRAME, is still expressed and will continue to play its regulatory role.

In conclusion, our study elucidates the regulatory role of the antisense lncRNA PRAME-AS in controlling PRAME transcript levels. We further demonstrate that MZF1 and a CpG island contribute significantly to the modulation of both PRAME-AS and PRAME expression. These findings suggest that the regulatory axis involving PRAME-AS, MZF1, and epigenetic elements such as CpG islands plays a pivotal role in PRAME regulation in cancers, highlighting PRAME-AS as a potential key contributor to tumor processes.

Minor comments:

Tables 1 & 2 can be added to supplementary information.

Response: We appreciate the thoughtful suggestion of the reviewer; however, we think that including these two tables in the main manuscript would provide readers with better access to information on primers and plasmid constructs."

Reviewer 4:

Hosseininia and Dehghani investigated the role of the antisense lncRNA of the PRAME gene (PRAME-AS) in PRAME regulation and cell phenotypes. The authors revealed that i) PRAME-AS knockout decreased PRAME mRNA levels by approximately 34%, ii) cell migration, viability, and stemness were decreased in PRAME-AS knockout cells compared

to control cells, iii) MZF1 increased the expression of PRAME-AS and PRAME transcripts, and iv) DNA hypermethylation of PRAME-AS upstream downregulated the expression of PRAME-AS and PRAME transcripts.

The authors concluded that PRAME-AS regulates PRAME at the transcriptional level. However, the data provided are insufficient to support this conclusion.

Major Comments:

1. Although the authors used HEK293 cells to investigate the effects of PRAME-AS on cell stemness, these cells were not optimal for the stemness assay due to their insufficient colony size for demonstrating differences. Furthermore, the function of PRAME is well-established in cancer cells. The authors should use at least one cancer cell line in addition to HEK293 cells. The utilization of more than two cell lines would be preferable for generalizing their findings.

Response: We thank the reviewer for this point. We have been able to detect and record significant differences in colony size in both the colony formation and soft-agar assays (Fig. 3). In addition, we did these experiments in three technical and two experimental (biological) replicates with a large number (n) of studied colonies in each group. For example, for colony size determination of soft agar assay (to evaluate stemness), we measured the diameter of a total of 145 colonies in PRAME-AS gene knockout cells in comparison with 181 colonies which were studied in wild-type cells, in two biological (experimental) and three technical replicates.

Regarding the use of a different cell line, we tried to use A375 melanoma cells. However, due to the much lower transfection efficiency of these cells, we were not able to achieve knockout cells. Additionally, the transcript levels of the PRAME-AS lncRNA gene were undetectable in these wild-type cells. To obtain reliable results that were not affected by limitations, we needed high transfection efficiency for all experiments, including transient modifications to hypermethylate CpG islands.

2. The authors should investigate the effect of PRAME-AS on cell growth. The number of control and PRAME-AS knockout cells should be counted from the initial day of culture until day 3 or 5.

Response: We thank the reviewer for this suggestion. We did not provide data on the cell growth curve since we were not able to detect any differences in the growth curve of the two groups of PRAME-AS wildtype and knockout cells after 3 days. Rather, to evaluate cell proliferation in the colony formation assay, the number and diameter of colonies were analyzed after 9 days, and in the soft agar assay, colonies were analyzed after 7 days (Fig 3B).

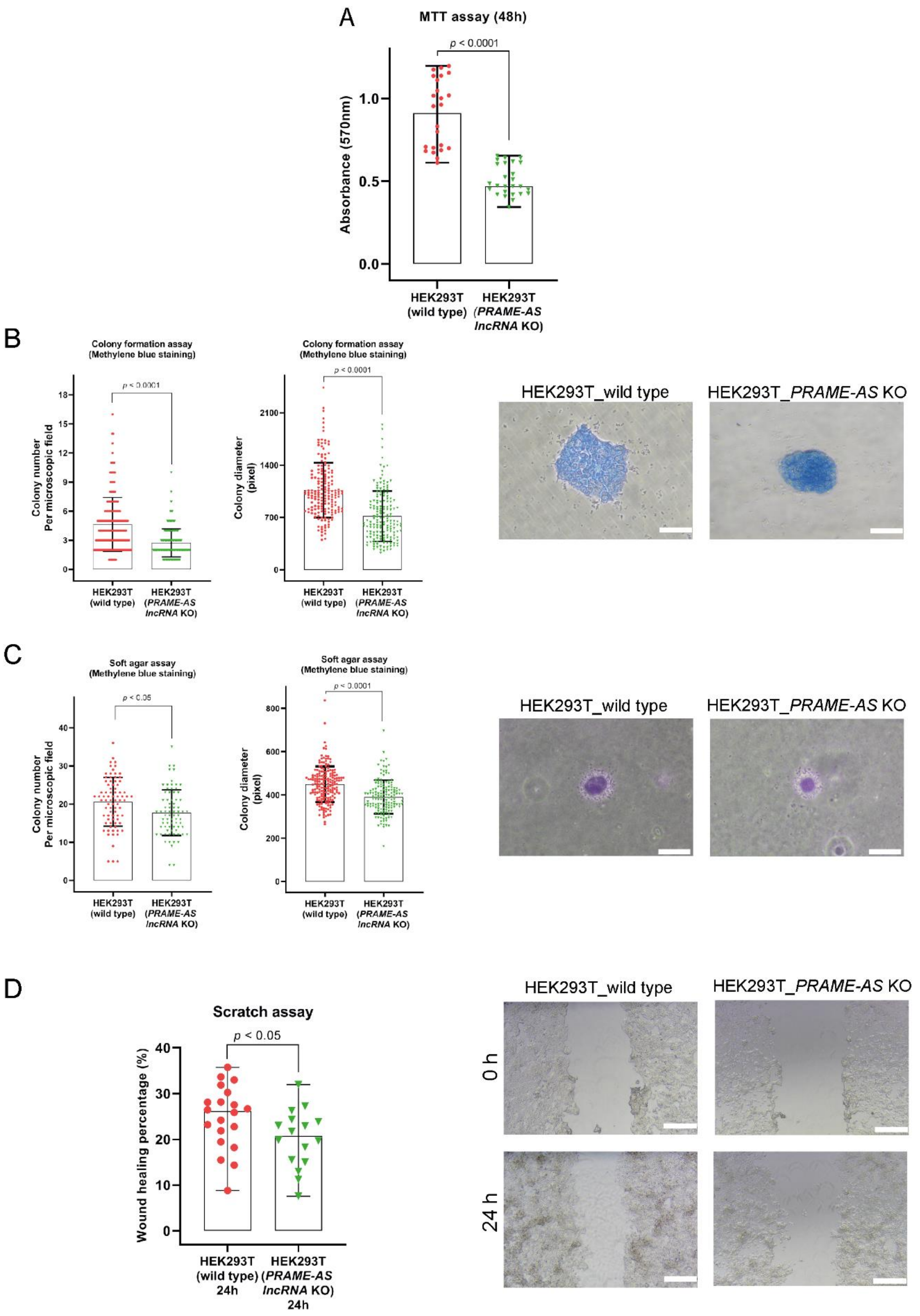
A
MTT assay (48h)
p < 0.0001
Absorbance (570nm)
HEK293T (wild type)
HEK293T (PRAME-AS lncRNA KO)
B
Colony formation assay (Methylene blue staining)
p < 0.0001
Colony number Per microscopic field
Colony diameter (pixel)
HEK293T_wild type
HEK293T_PRAME-AS KO
C
Soft agar assay (Methylene blue staining)
p < 0.05
p < 0.0001
D
Scratch assay
p < 0.05
Wound healing percentage (%)
0 h
24 h
HEK293T (wild type) 24h
HEK293T (PRAME-AS lncRNA KO) 24h

3. Despite being statistically significant, the observed reduction in PRAME mRNA levels and the diminished stemness and migratory capabilities resulting from PRAME-AS knockout appear to be minimal. These subtle changes may not result in substantial biological effects. The authors should provide an explanation for the modest decrease in PRAME mRNA and elucidate why there are only slight differences between the wild-type cells and those with PRAME-AS knockout in both the soft agar and scratch assays (Figures 2G, 3C, and 3D).

Response: As was explained in "Introduction" and discussed in "Discussion", different regulatory pathways have been reported to be involved in the PRAME transcription. Different cellular functions are regulated by various genetic and epigenetic factors and regulatory networks. Since in this study, we manipulated only the PRAME-AS gene, we cannot expect to observe considerable changes in PRAME expression and cellular functions. As an example, in the PRAME-AS knockout cells, MZF1, which is a positive regulator of PRAME, is still expressed and will continue to play its regulatory role. We have added a section to "Discussion" to emphasize this point.

4. Although the authors avoided CpG island on the primers for bisulfite sequencing (page 10, line 162), DNA methylation of CpG island is crucial for the regulation of gene expression. Primers should be designed to analyze CpG methylation on CpG islands.

Response: We did not avoid analyzing the CpG island. In contrast, we explored the island for methylation of CpG dinucleotides. In the revised version, we have added explanations to "Materials and Methods" to clarify this point. We explained that based on the criteria for bisulfite PCR, it is necessary to design primers that flank the CpG-containing sequences. The designed primers in this study amplified a product that encompassed the CpGs, allowing us to analyze them after bisulfite conversion (Fig. 5D).

5. Figure 6A should be carefully edited for the following reasons:

a) Although the authors stated that H3K27ac data of the PRAME promoter was from UCSC Genome Browser (page 25, line 495), the observed H3K27ac peak differed from the current information available on the UCSC Genome Browser (https://genome.ucsc.edu/cgi-bin/hgTracks?db=hg38&lastVirtModeType=default&lastVirtModeExtraState=&virtModeType=default&virtMode=0&nonVirtPosition=&position=chr22%3A22556888%2D22562353&hgsid=2476779099_S0v0GSEFx5vKwQS2lhUHcGN4aBHD).

Response: We thank the reviwer for this point. The appearance of the H3K27ac peak was corrected based on the UCSC Genome Browser and the above link.

b) The positions of CpG island are based on a previous study (Schenk T et al, ref#22). However, this is the predicted position, and the current official annotation of CpG island locations can be found in the UCSC Genome Browser.

Response: The positions of CpG dinucleotides in the CpG island reported by Schenk T et al. (ref#22) are the results of genomic sequencing in various human patient samples and cell lines. In addition, the majority of CpG island in the UCSC Genome Browser overlaps the region reported by Schenk T et. al. In this region, we selected those CpG dinucleotides that based on the above paper, are more likely to affect PRAME expression depending on their methylation status.

c) It is unclear how to generate the continuous peak of PRAME promoter activity from the intermittent data obtained through the luciferase assay.

Response: In the new Fig. 6, we have corrected the presentation of PRAME promoter activity.

6. The authors should carefully edit the entire manuscript:
a) The Discussion section needs revision to eliminate redundancies and excessive length; for examples, the detailed descriptions of other antisense lncRNA are not necessary (page 27, lines 534 – 548), the detailed information of MZF1 (page 29, lines 574 – 595) should be shorten because the regulation of PRAME by MZF1 has been reported in previous papers (Lee et al, ref#23 etc.), repeated description of the results are not necessary (page 30, lines 612 – 616).
Response: We have edited the entire manuscript, including figures and tables. We also eliminated redundant explanations throughout the manuscript. These changes are shown in the version with tracked changes.

b) "CPG" should be changed to "CpG" throughout the manuscript.
Response: We thank the reviewer for this careful note. We have done this correction throughout the manuscript.
