# Supplementary material for: PRAME-AS lncRNA, regulated by MZF1, modulates PRAME expression and cell stemness
Source: PLoS One. 2025 Sep 17;20(9):e0331190. doi: 10.1371/journal.pone.0331190 (PMC12443320; doi:10.1371/journal.pone.0331190)
Supplement: Attachment — Submitted filename: Response_to_reviewers_auresp_2.pdf [file pone.0331190.s015.pdf]

# RESPONSE TO REVIEWERS –(PONE-D-25-03086R1)

We would like to express our sincere gratitude to the reviewers for their time and thoughtful comments on our manuscript. Below, we provide a point-by-point response to Reviewer #2's comments. The unedited comments from the reviewer are presented in italics.

**Reviewer #2: Minor comments for Hosseininia & Dehghani:**

*1. Figure 2A spelling: "PRAM-AS"*

Response: We thank the reviewer for pointing this out. We have corrected this typo, along with a few others that were found in other figures.

*2. Wording regarding unidirectionality – PRAME-AS transcription appears to be dependent on PRAME transcription. I don't think it is correct to say that "PRAME promoter operates unidirectionally, regulating only PRAME transcription". PRAME promoter clearly regulates PRAME-AS as well through bidirectional transcription. Perhaps more accurate to say that PRAME-AS strand promoter region is insufficient for transcription (based on reporter expression) without PRAME transcription (the PRAME strand) to drive its expression.*

Response: We agree that the potential involvement of the PRAME promoter in the transcriptional regulation of PRAME should be taken into account. This possibility has already been discussed in the "Discussion" section:

However, our reporter assay that includes the PRAME locus regulatory region did not reveal a considerable promoter function in the PRAME-AS direction (Fig 6). In support of our findings, a recent study reported that a ~300-nucleotide common regulatory region with signatures of a bidirectional promoter for the genes LINC00882 and DUBR, lost its promoter activity when upstream regions were also cloned next to this common region [66]. Thus, one possible explanation for our findings is that the promoter activity of the defined region may have been negatively affected by additional upstream sequences.

Per the reviewer's suggestion, we have revised the sentence in the "Abstract", "Results" and Figure 6 legend as follows:

Abstract and Results

Based on our EGFP reporter assay, we conclude that the regulatory region of the PRAME locus—without PRAME transcription—is insufficient to drive transcription of PRAME-AS in the antisense direction.

Figure 6 legend

The PRAME locus regulatory region regulates transcription of a reporter gene only in the PRAME direction

*3. In the discussion it is mentioned that a possible regulatory mechanism may involve direct interactions between PRAME and PRAME-AS via RNA-RNA duplex. A BLAST search of spliced PRAME-AS compared to PRAME entire transcript (exon and intron) seems to indicate that ~100nt of PRAME-AS exon 3 has complementarity with PRAME intron. If RNA-RNA duplex is mentioned as a possibility, then evidence or lack of evidence of this through sequence search should be done.*

Response: We appreciate the reviewer's important point. To examine potential interactions between spliced PRAME-AS and PRAME mRNA, we used several tools, including LncTar, LncRRIsearch, ENCORI, RNAInter, and NPInter v5.0. However, none of these tools predicted any interactions. We also performed BLAST analysis and found that exon 3 of PRAME-AS shares sequence similarity with a 256-base region within intron 3 of the PRAME gene. Additionally, exon 1 of PRAME-AS shows similarity to a 65-base region in the 5′ UTR of the PRAME gene, located 77 bases upstream of the transcription start site (TSS). However, we note that BLAST analysis identifies sequence similarity rather than complementarity.

Thus, considering the above explanations and the multitude of mechanisms through which lncRNAs can influence mRNA levels, we believe that concluding the “Discussion” section with a sentence indicating that further experiments are needed to elucidate the underlying mechanisms would be appropriate.
